# Who uses new walking and cycling infrastructure and how? Longitudinal results from the UK iConnect study^☆^

**DOI:** 10.1016/j.ypmed.2013.07.007

**Published:** 2013-11

**Authors:** Anna Goodman, Shannon Sahlqvist, David Ogilvie, on behalf of the iConnect consortium

**Affiliations:** a Medical Research Council Epidemiology Unit and UKCRC Centre for Diet and Activity Research (CEDAR), University of Cambridge, Box 296, Institute of Public Health, Forvie Site, Robinson Way, Cambridge, CB2 0SR, UK; b Faculty of Epidemiology and Population Health, London School of Hygiene and Tropical Medicine, London, WC1E 7HT, UK; c Centre for Physical Activity and Nutrition Research (C-PAN), School of Exercise and Nutrition Sciences, Deakin University, 221 Burwood Highway, Burwood, VIC 3125, Australia

**Keywords:** Walking, Cycling, Built environment, Infrastructure

## Abstract

**Objective:**

To examine how adults use new local walking and cycling routes, and what characteristics predict use.

**Methods:**

1849 adults completed questionnaires in 2010 and 2011, before and after the construction of walking and cycling infrastructure in three UK municipalities. 1510 adults completed questionnaires in 2010 and 2012. The 2010 questionnaire measured baseline characteristics; the follow-up questionnaires captured infrastructure use.

**Results:**

32% of participants reported using the new infrastructure in 2011, and 38% in 2012. Walking for recreation was by far the most common use. In both follow-up waves, use was independently predicted by higher baseline walking and cycling (e.g. 2012 adjusted rate ratio 2.09 (95% CI 1.55, 2.81) for > 450 min/week vs. none). Moreover, there was strong specificity by mode and purpose, e.g. baseline walking for recreation specifically predicted walking for recreation on the infrastructure. Other independent predictors included living near the infrastructure, better general health and higher education or income.

**Conclusions:**

The new infrastructure was well-used by local adults, and this was sustained over two years. Thus far, however, the infrastructure may primarily have attracted existing walkers and cyclists, and may have catered more to the socio-economically advantaged. This may limit its impacts on population health and health equity.

## Highlights

• In three UK settings, new walking and cycling routes were well-used by local adults.
• Despite aiming to ‘connect places’, routes were used more for recreation than for transport.
• Baseline walking and cycling strongly predicted route use at 1 and 2 year follow-up.
• Thus far, the routes may have mainly attracted existing walkers and cyclists.

## Introduction

In the past two decades, promoting walking and cycling has gained increased policy attention in multiple sectors including health, transport and climate change (Chief Medical Officers of England, 2011; Department of Health and Department for Transport, 2010; THE PEP, 2009; WHO, 2002). It is increasingly recognised that creating a supportive built environment may play a crucial role in enabling the success of individual-level interventions (Giles-Corti, 2006) and in promoting enduring population behaviour change (Butland et al., 2007; Institute of Medicine and National Research Council of the National Academies, 2009; NICE, 2008).

Nevertheless, several reviews have highlighted the paucity of controlled, longitudinal studies evaluating new infrastructure for walking or cycling (e.g. Krizek et al., 2009; McCormack and Shiell, 2011; NICE, 2008; Pucher et al., 2009) and many of the studies that do exist have used repeat cross-sectional rather than cohort designs (Ogilvie et al., 2007; Yang et al., 2010). These studies cannot prospectively determine the individual, household or geographic predictors of using new infrastructure. Given that inactive people derive the most benefit from additional physical activity (US Department of Health and Human Services, 1996; Woodcock et al., 2011), new infrastructure would be expected to generate greater public health gains if it attracted new walking or cycling trips rather than existing walkers and cyclists (Ogilvie et al., 2007; Yang et al., 2010), but we know of no study examining associations between use and baseline activity levels. From an equity perspective, it may also be important to examine the socio-demographic predictors of use, and so evaluate whether the infrastructure meets the needs of all groups (Marmot, 2010; NICE, 2008, 2012).

In addition to identifying *who* uses new infrastructure, it is also useful to examine *what* it is used for because this may affect its health and environmental impacts. For example, cycling is typically a higher intensity activity than walking and so may have a greater effect upon physical fitness (Yang et al., 2010). Similarly, transport trips may confer greater environmental benefits than recreational trips, because active travel seems to substitute for motor vehicle use whereas recreational walking may involve it (Goodman et al., 2012).

Finally, whereas most previous longitudinal studies included only a single follow-up wave (Ogilvie et al., 2007; Yang et al., 2010), comparing results across multiple waves may provide insights into changing patterns of use or a changing profile of users. This may be important for understanding effects beyond the immediate post-intervention period: for example, although early adopters may be those who are already physically active, social modeling may subsequently encourage use by more inactive individuals (Ogilvie et al., 2011).

This paper therefore aims to examine and compare patterns of using high-quality, traffic-free walking and cycling routes over one- and two-year follow-up periods. Specifically, we examine the journey purposes for which the infrastructure was used and the modes by which it was used. We also examine the individual and household predictors of use.

## Methods

### Intervention, study sites and sample

Led by the sustainable transport charity Sustrans, the Connect2 initiative is building or improving walking and cycling routes at multiple sites across the United Kingdom (map in Supplementary material). Each Connect2 site comprises one flagship engineering project (the ‘core’ project) plus improvements to feeder routes (the ‘greater’ project). These projects are tailored to individual sites but all embody a desire to create new routes for “everyday, local journeys by foot or by bike” (Sustrans, 2010).

**Fig. 1:**
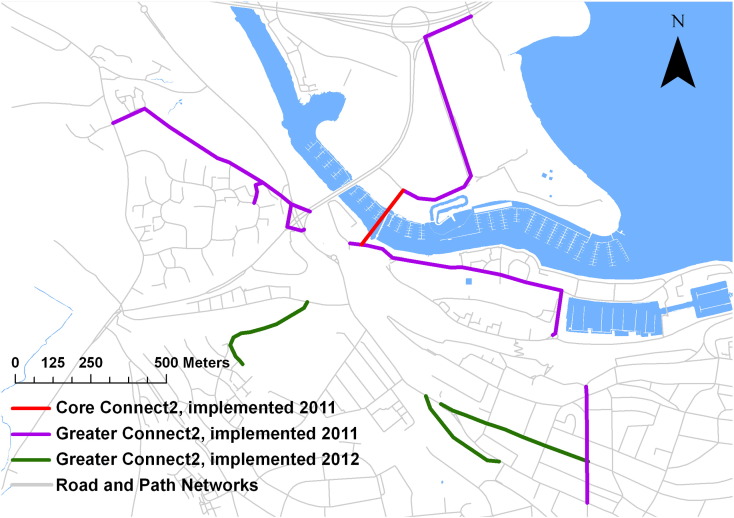
‘Core’ and ‘greater’ Connect2 projects at the Cardiff study site. Purple lines show the sections of the greater Connect2 network which were operational at the time of both the 2011 and the 2012 surveys; green lines show the sections of the network only operational at the time of the 2012 survey. See electronic supplement for equivalent maps of Southampton and Kenilworth, and for the location within the United Kingdom of these three study sites. Contains Ordnance Survey data © Crown copyright and database right 2011.

The independent iConnect research consortium (www.iconnect.ac.uk) was established to evaluate the travel, physical activity and carbon impacts of Connect2 (Ogilvie et al., 2011, 2012). As previously described in detail (Ogilvie et al., 2012), three Connect2 projects were selected for detailed study according to criteria including implementation timetable, likelihood of measurable population impact and heterogeneity of overall mix of sites. These study sites were: Cardiff, where a traffic-free bridge was built over Cardiff Bay; Kenilworth, where a traffic-free bridge was built over a busy trunk road; and Southampton, where an informal riverside footpath was turned into a boardwalk (Ogilvie et al., 2012). None of these projects had been implemented during the baseline survey in April 2010. At one-year follow-up, most feeder routes had been upgraded and the core projects had opened in Southampton and Cardiff in July 2010. At two-year follow-up, almost all feeder routes were complete and the core Kenilworth project had opened in September 2011. Fig. 1 illustrates the traffic-free bridge built in Cardiff (the ‘core’ project in this setting) plus the feeder routes implemented in 2010 and 2011 (the ‘greater’ network).

The baseline survey used the edited electoral register to select 22,500 adults living within 5 km road network distance of the core Connect2 projects (Ogilvie et al., 2012). In April 2010 potential participants were posted a survey pack, which 3516 individuals returned. These 3516 individuals were posted follow-up surveys in April 2011 and 2012; 1885 responded in 2011 and 1548 in 2012. After excluding individuals who had moved house, the one-year follow-up study population comprised 1849 participants (53% retention rate, 8% of the population originally approached) and the two-year study population comprised 1510 (43% retention, 7% of the original population). The University of Southampton Research Ethics Committee granted ethical approval (CEE200809-15).

### Baseline characteristics

Table 1 presents the baseline characteristics examined as predictors of Connect2 use. Past-week walking and cycling for transport were measured using a seven-day recall instrument (Goodman et al., 2012; Ogilvie et al., 2012) while past-week recreational walking and cycling were measured by adapting the short form of the International Physical Activity Questionnaire (Craig et al., 2003). Most other predictors were similarly self-reported, including height and weight from which we calculated body mass index (categorised as normal/overweight/obese). The only exception was the distance from the participant's home to the nearest access point to a completed section of the greater Connect2 infrastructure (calculated separately in 2011 and 2012 to reflect ongoing upgrades: Fig. 1). This was calculated in ArcGIS 9 using the Ordnance Survey's Integrated Transport Network and Urban Path layers, which include the road network plus traffic-free or informal paths. For ease of interpretation, we reverse coded distance from the intervention to generate a measure of proximity – i.e. treating those living within 1 km as having a higher proximity than those living over 4 km away (Table 1).

### Awareness and use of Connect2

At follow-up, participants were given a description of their local Connect2 project and asked “Had you heard of the [Connect2 infrastructure] before completing this survey?” (yes/no) and “Do you use the [Connect2 infrastructure]?” (yes/no). Participants reporting using Connect2 were then asked whether they (a) walked or (b) cycled on Connect2 for six journey purposes (commuting for work, travel for education, travel in the course of business, shopping or personal business, travel for social or leisure activities, and recreation, health or fitness).

### Statistical analyses

We examined the predictors of (i) Connect2 awareness and (ii) Connect2 use using Poisson regression with robust standard errors (Zou, 2004). We initially adjusted analyses only for age, sex and study site, and then proceeded to multivariable analyses. Missing data across explanatory and outcome variables ranged from 0 to 8.1% per variable, and were imputed using multiple imputation by chained equations under an assumption of missing at random. To allow for potential correlations between participants living in the same neighbourhood, robust standard errors were used clustered by Lower Super Output Area (average population 1500). Statistical analyses were conducted in 2012–2013 using Stata 11.

## Results

### Characteristics of study participants

Comparisons with local authority and national data suggested that participants included fewer young adults than the general population (e.g. 7% in the two-year sample vs. 26% of adults locally) and were also somewhat healthier, better-educated and less likely to have children. Otherwise the study population appeared to be broadly representative in its demographic, socio-economic, travel and activity-related characteristics (see Supplementary material). Retention at follow-up did not differ with respect to proximity to the intervention or baseline levels of walking and cycling (see Supplementary material). The one- and two-year study samples had very similar characteristics (Table 1), and all findings were unchanged in sensitivity analyses restricted to those who provided data at both time points.

### Awareness and use of Connect2

Awareness and use of Connect2 were fairly high at one-year follow-up, with 32% reporting using Connect2 and a further 32% having heard of it. At two-year follow-up these proportions had risen slightly to 38% and 35%. Among those taking part in both follow-up waves, the correlation between use at one and two years was 0.62, with (for example) 82% of those who used it at one year reporting also using it at two years (Table 2 and Supplementary material). Correlations for specific types of use were generally also fairly high, ranging from 0.35 to 0.76.

The average number of types of Connect2 use reported by users was 1.96 at one-year follow-up and 1.97 at two-year follow-up. In both follow-up waves, walking for recreation was by far the most commonly reported type of Connect2 use, followed by cycling for recreation, walking for transport and cycling for transport (Table 2). This predominant use of Connect2 for walking was unsurprising given that walking was much more common in general than cycling among our participants. If anything, use of Connect2 for cycling was more common than might have been expected from baseline measures of past-week cycling. For example, at baseline around five times more participants reported doing any walking in the past week than reported any cycling (83% vs. 16%), whereas at follow-up ‘only’ around twice as many reported walking on Connect2 as reported cycling.

In contrast, the dominance of recreational use of Connect2 could not be explained in this way, as baseline levels of walking or cycling were similar across recreation and transport purposes, with 65% vs. 66% reporting any in the past week. Among those who used Connect2 for transport, the most frequently reported journey purposes were social and leisure trips, followed by shopping and personal business. Only 8% of Connect2 users (11% of users who were in employment) reported using Connect2 for work or business at one-year follow-up, and 9% (13% of those in employment) at two years.

### Predictors of awareness and use of Connect2

**Fig. 2:**
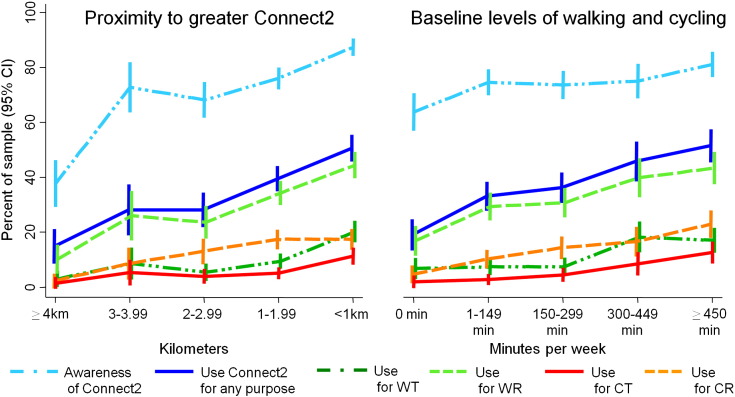
Effect of proximity to Connect2 and baseline walking and cycling upon awareness and different types of Connect2 use at two-year follow-up. CR = cycling for recreation; CT = cycling for transport; CI = confidence interval, km = kilometers; min = minutes; WR = walking for recreation; WT = walking for transport. Results based on 1510 British adults participating in 2010 and 2012: Results very similar at one-year follow-up, see the Supplementary material.

Table 3 shows the predictors of using Connect2 for any purpose. In general, the associations at one- and two-year follow-up were very similar. Use was highest in Cardiff and lowest in Southampton (Table 3). The other strongest predictors were living closer to Connect2 and higher baseline walking and cycling. These variables both showed dose-response associations of a very similar magnitude at one and two years, and were also associated with awareness of Connect2 and with the various different modes and purposes of Connect2 use (Fig. 2). With respect to baseline walking and cycling, these associations were highly mode- and purpose-specific: when past-week walking and cycling for transport and recreation were entered as four separate variables, the baseline behaviour in question was almost always the strongest predictor and was usually the only significant predictor (e.g. past-week walking for transport specifically predicted walking for transport on Connect2: see Supplementary material). All findings were very similar in sensitivity analyses using proximity to the core rather than to the greater Connect2 project.

Other strong, independent predictors of Connect2 use were non-student status and household bicycle access, although the latter association was attenuated somewhat after adjusting for baseline walking and cycling. Higher income and education also predicted Connect2 use at both follow-up waves in minimally-adjusted analyses, although only one of these was ever significant in adjusted analyses. Older age (> 65 years), obesity and poorer health all predicted lower Connect2 use in minimally-adjusted analyses. However, these associations were generally attenuated to the null after adjusting for other characteristics, particularly baseline walking and cycling, and/or were not replicated across follow-up waves. The associations were generally similar across the three study sites, with most variables showing no consistent evidence (p < 0.1) of interaction across the two timepoints. The only exception was consistent, weak evidence (0.02 ≤ p ≤ 0.03 for interaction) that men were more likely to use Connect2 in Southampton but not in the other two sites (e.g. rate ratio 1.44 (95%CI 1.03, 2.02) for men vs. women in Southampton in 2012, versus point estimates of 1.03 in Cardiff and 0.97 in Kenilworth).

The Supplementary material presents the predictors of using Connect2 for walking and cycling for transport and recreation, modelled as four separate outcomes. The findings were generally similar to those presented in Table 3, except that bicycle access and, to a lesser extent, higher education were more strongly associated with using Connect2 for cycling than for walking.

## Discussion

### Sustained usage, but dominance of recreational trip purposes

The stated aim of Connect2 was to serve local populations and provide new routes for everyday journeys (Sustrans, 2010). Some success is indicated by the fact that a third of participants reported using Connect2 and a further third had heard of it, with higher awareness and use among residents living closer to the projects. The slight increase in awareness and use by two-year follow-up suggests that these findings do not simply reflect temporary publicity surrounding the Connect2 opening or a novelty effect of wanting to ‘try it out’ once.

Yet despite Connect2's emphasis on “connecting places”, we replicated previous research on American trails (Price et al., 2012, 2013) in finding that many more participants used Connect2 for recreational than for transport purposes. This did not simply reflect lower total walking and cycling for transport among participants, nor does the built environment appear to matter less for transport than for recreation in general (McCormack and Shiell, 2011; Owen et al., 2004). Instead the dominance of recreational uses may reflect the fact that these Connect2 projects did not constitute the comprehensive *network*-*wide* improvements that may be necessary to trigger substantial modal shift (NICE, 2008). In other words, although Connect2 provided all local residents with new (and apparently well-used) locations for recreation, it may not have provided most residents with practical new routes to the particular destinations they needed to reach. This interpretation is consistent with the observation that among those who did use Connect2 for transport, many more reported making shopping and leisure trips than commuting or business trips; the former may typically afford more opportunity to choose between alternative destinations than the latter.

### Broad socio-demographic appeal, but higher use in more advantaged and active groups

Connect2 seemed to have a broad demographic appeal, with relatively little variation in use by age, gender, ethnicity or household composition. Higher education or income did, however, independently predict Connect2 use, a finding consistent with one (Brownson et al., 2000) but not all (Brownson et al., 2004; Merom et al., 2003) previous studies. This association was particularly strong for cycling suggesting that, at least in this setting and in the short term, Connect2 may not reduce the existing or emerging socio-economic gradients in cycling in the UK (Goodman, in press; Marmot, 2010).

Connect2 use was strongly predicted by higher pre-intervention levels of walking and cycling, an association which showed a marked specificity by mode and purpose. This suggests that many users may have changed *where* they walked or cycled without changing *what* they were doing. Such displacement would be consistent with previous studies reporting that most users of new off-road ‘trails’ had been walking or cycling prior to their construction (Burbidge and Goulias, 2009; Gordon et al., 2004). Our evaluation builds on those studies by showing the effect was stable over two years, with no suggestion that previously less active individuals formed a higher proportion of users over time. It is possible that attracting less active individuals may require larger infrastructure changes (e.g. network-wide improvements) or more time (e.g. with improved infrastructure being necessary but not sufficient, and with behaviour change being triggered by subsequent individual life events) (Christensen et al., 2012; Giles-Corti and Donovan, 2002; Jones and Ogilvie, 2012). On the other hand, even among the least active individuals the proportion using Connect2 was not trivial (e.g. 17–19% among those reporting no past-week activity at baseline), indicating some potential for such infrastructure to appeal to users of all activity levels.

### Strengths and limitations

Strengths of this study include its cohort design and population-based sampling, which allowed us to address novel substantive questions such as who used the new infrastructure. Nevertheless, there are also some key limitations. One is the potential for selection bias: given the low response rate, the study population cannot be assumed to be representative. Yet although on average older than the general population, participants generally appeared fairly similar in their demographic, socio-economic and travel-related characteristics; and retention at follow-up was not predicted by proximity to the intervention or baseline physical activity, the two strongest predictors of infrastructure use. A second important limitation is that, for each mode and purpose, we measured only whether each participant used Connect2, not the frequency of use. It is plausible that frequent and habitual transport journeys such as commuting form a higher proportion of Connect2 *trips* than the 7% of Connect2 users who reported using the infrastructure to travel to work. This would be consistent with a previous intercept survey on the traffic-free routes making up the National Cycle Network, which found a more equal balance of trips made for transport (43%) and trips made for recreation (57%) (Lawlor et al., 2003).

## Conclusions

At one- and two-year follow-up, Connect2 infrastructure was well-used and therefore has the potential to encourage environmentally sustainable physical activity in local communities. Thus far, however, its users have tended to be more physically active and socio-economically advantaged residents, which may limit its impacts on overall population health and health equity. We therefore intend to examine in future analyses the extent to which these relatively high levels of infrastructure use translate into overall increases in walking, cycling and physical activity, and into overall decreases in motorised travel and associated carbon emissions. We also intend to examine which particular changes in the Connect2 routes encourage use. This will involve integrating additional quantitative and qualitative research conducted within the broader iConnect program, and will capitalize on the observed heterogeneity between study sites in intervention characteristics and in levels of use. Through close attention to mechanisms and contexts, we hope to examine not only whether environmental interventions like Connect2 ‘work’, but also why they do or do not work, for whom and in what circumstances (Ogilvie et al., 2011).

## Conflict of interest statement

The authors declare that there are no conflicts of interest.

## Figures and Tables

**Table 1:** Participants' characteristics at baseline.

Domain	Variable	Level	N (%) in one-year sample	N (%) in two-year sample
Geographic	Site	Southampton	523 (28%)	425 (28%)
Cardiff	596 (32%)	487 (32%)
Kenilworth	730 (39%)	598 (40%)
Proximity of home to greater Connect2 (km)	≥ 4	178 (10%)	144 (10%)
3–3.99	137 (7%)	106 (7%)
2–2.99	291 (16%)	229 (15%)
1–1.99	631 (34%)	490 (33%)
< 1	612 (33%)	541 (36%)
Demographic	Sex	Female	1006 (54%)	857 (57%)
Male	843 (46%)	653 (43%)
Age (years) at baseline	18–34	241 (13%)	144 (10%)
35–49	379 (21%)	300 (20%)
50–64	607 (33%)	532 (35%)
65–89	616 (33%)	530 (35%)
Ethnicity	White	1771 (97%)	1460 (97%)
Non-White	64 (3%)	45 (3%)
Any child under 16	No	1547 (84%)	1276 (85%)
Yes	301 (16%)	234 (16%)
Socio-economic status and car and bicycle access	Highest educational level	Tertiary or equivalent	715 (39%)	590 (39%)
Secondary school †	622 (34%)	490 (33%)
None or other	500 (27%)	425 (28%)
Annual household income	>£40,000	584 (34%)	451 (32%)
£20,001–40,000	550 (32%)	469 (33%)
≤£20,000	577 (34%)	488 (35%)
Employment status	Working	939 (51%)	740 (49%)
Student	48 (3%)	25 (2%)
Retired	710 (38%)	609 (40%)
Other	152 (8%)	134 (9%)
Any car in household	No	247 (13%)	215 (14%)
Yes	1599 (87%)	1290 (86%)
Any adult bicycle in household	No	768 (45%)	620 (45%)
Yes	948 (55%)	768 (55%)
Health	Weight status	Normal/underweight	875 (50%)	702 (49%)
Overweight	641 (36%)	534 (37%)
Obese	244 (14%)	201 (14%)
General health	Excellent/good	1437 (79%)	1168 (78%)
Fair/poor	388 (21%)	324 (22%)
Long-term illness or disability that limits daily activities	No	1295 (75%)	1046 (74%)
Yes	441 (25%)	374 (26%)
Walking and cycling	Time spent walking or cycling in past week (minutes)	None	295 (16%)	238 (16%)
1–149	479 (26%)	384 (25%)
150–299	415 (22%)	355 (24%)
300–449	270 (15%)	218 (14%)
≥ 450	390 (21%)	315 (21%)

km = kilometers. † British ‘A’ Levels, GCSEs or equivalent. Results based on 1849 British adults participating in 2010 and 2011, and 1510 participating in 2010 and 2012: numbers add to fewer than the total number of participants for some variables due to missing data. Chi-squared tests provided no evidence (all p > 0.16) for a difference in the distribution of characteristics between the one- and two-year samples, except that the age distribution was (unsurprisingly) slightly older at two-year follow-up.

**Table 2:** Proportion of study population reporting using Connect2 for different purposes.

		% of full sample reporting behaviour		% Connect2 users reporting behaviour		Correlation across years †
		One year	Two years	One year	Two years
Walking	Any transport	11%	12%	35%	32%	.51
Social/leisure	8%	8%	26%	22%	.48
Shopping/personal	6%	6%	18%	15%	.46
To work	1%	1%	4%	3%	.38
For education	< 1%	< 1%	2%	1%	.41
For business	< 1%	< 1%	2%	2%	.49
Recreation	27%	32%	84%	85%	.60
Any walking	29%	35%	92%	91%	.61
Cycling	Any transport	5%	7%	17%	18%	.60
Social/leisure	4%	5%	11%	13%	.40
Shopping/personal	2%	2%	7%	6%	.41
To work	1%	2%	5%	5%	.61
For education	< 1%	< 1%	< 1%	1%	.76
For business	< 1%	< 1%	1%	2%	.35
Recreation	12%	15%	37%	39%	.65
Any cycling	13%	16%	39%	43%	.66
Use Connect2 for any purpose		32%	38%	100%	100%	.62

Results based on 1826 British adults from the one-year sample in 2011 (excluding 1.2% with missing data), and 1490 from the two-year sample in 2012 (excluding 1.3% with missing data). † Pearson correlation between reporting behaviour at one and two years, among those taking part at both time points (N = 1235). The Supplementary material includes the numbers of individuals underlying these percentages and correlations.

**Table 3:** Predictors of using Connect2 for any purpose at one- or two-year follow-up.

Baseline characteristics	Level	One-year sample (N = 1849)			Two-year sample (N = 1510)		
% users	Minimally-adjusted RR (95% CI)	Multivariable RR (95% CI)	% users	Minimally-adjusted RR (95% CI)	Multivariable RR (95% CI)
Site	Southampton	20%	1***	1***	23%	1***	1***
Cardiff	49%	2.48 (1.86, 3.32)	2.26 (1.75, 2.91)	53%	2.37 (1.83, 3.06)	2.10 (1.70, 2.60)
Kenilworth	28%	1.45 (1.04, 2.01)	1.57 (1.19, 2.08)	37%	1.70 (1.29, 2.23)	1.70 (1.38, 2.09)
Proximity of home to greater Connect2 (km)	≥ 4	12%	1***	1***	16%	1***	1***
3–3.99	22%	1.57 (0.94, 2.63)	1.58 (0.97, 2.57)	28%	1.63 (1.01, 2.66)	1.54 (0.95, 2.51)
2–2.99	25%	2.13 (1.24, 3.66)	2.06 (1.26, 3.37)	27%	1.88 (1.22, 2.88)	1.79 (1.22, 2.63)
1–1.99	31%	2.86 (1.73, 4.73)	2.71 (1.67, 4.40)	37%	2.80 (1.90, 4.13)	2.59 (1.81, 3.71)
< 1	46%	3.83 (2.33, 6.31)	3.62 (2.27, 5.80)	52%	3.54 (2.37, 5.29)	3.38 (2.35, 4.87)
Sex	Female	31%	1	1	37%	1*	1
Male	34%	1.11 (0.99, 1.25)	1.08 (0.96, 1.21)	40%	1.13 (1.01, 1.25)	1.07 (0.95, 1.19)
Age (years) at baseline	18–34	33%	1	1	40%	1*	1*
35–49	40%	1.22 (0.91, 1.65)	1.10 (0.89, 1.38)	45%	1.14 (0.90, 1.43)	0.87 (0.69, 1.10)
50–64	35%	1.05 (0.78, 1.42)	1.05 (0.83, 1.34)	43%	1.06 (0.85, 1.33)	0.96 (0.78, 1.19)
65–89	26%	0.78 (0.57, 1.08)	0.88 (0.65, 1.21)	29%	0.72 (0.56, 0.93)	0.74 (0.57, 0.95)
Ethnicity	White	33%	1	1	39%	1	1
Non-White	27%	0.85 (0.55, 1.30)	0.99 (0.68, 1.45)	25%	0.70 (0.41, 1.18)	0.83 (0.51, 1.33)
Any child under 16	No	31%	1	1	36%	1	1
Yes	42%	1.17 (0.96, 1.43)	1.04 (0.85, 1.27)	48%	1.16 (0.96, 1.41)	1.12 (0.94, 1.32)
Highest educational level	Tertiary	42%	1***	1*	49%	1***	1
Secondary	28%	0.68 (0.59, 0.79)	0.84 (0.73, 0.96)	34%	0.70 (0.60, 0.82)	0.86 (0.74, 1.00)
None or other	24%	0.65 (0.54, 0.79)	0.88 (0.75, 1.03)	28%	0.68 (0.57, 0.81)	0.92 (0.77, 1.10)
Annual household income	>£40,000	42%	1**	1	51%	1***	1*
£20–40,000	32%	0.83 (0.69, 1.00)	0.90 (0.75, 1.07)	39%	0.82 (0.71, 0.94)	0.89 (0.78, 1.02)
≤£20,000	24%	0.65 (0.52, 0.82)	0.82 (0.66, 1.03)	27%	0.62 (0.52, 0.74)	0.77 (0.64, 0.95)
Employment status	Working	37%	1**	1*	44%	1***	1**
Student	10%	0.33 (0.14, 0.75)	0.34 (0.15, 0.76)	8%	0.22 (0.06, 0.80)	0.20 (0.06, 0.68)
Retired	29%	1.13 (0.93, 1.37)	1.23 (1.01, 1.50)	34%	1.12 (0.94, 1.33)	1.17 (0.97, 1.40)
Other	28%	0.81 (0.64, 1.02)	0.96 (0.75, 1.23)	28%	0.66 (0.50, 0.87)	0.77 (0.59, 1.00)
Any car in household	No	23%	1*	1	27%	1*	1
Yes	34%	1.34 (1.04, 1.72)	1.07 (0.83, 1.37)	40%	1.31 (1.05, 1.63)	0.99 (0.80, 1.21)
Any adult bicycle in household	No	23%	1***	1***	27%	1***	1***
Yes	41%	1.74 (1.44, 2.11)	1.51 (1.26, 1.80)	48%	1.68 (1.43, 1.97)	1.45 (1.25, 1.70)
Weight status	Normal	33%	1	1	40%	1*	1
Overweight	33%	0.97 (0.84, 1.14)	1.07 (0.94, 1.22)	39%	0.95 (0.83, 1.10)	1.03 (0.90, 1.18)
Obese	27%	0.78 (0.63, 0.97)	1.02 (0.84, 1.24)	30%	0.72 (0.57, 0.92)	0.97 (0.77, 1.21)
General health	Excellent/good	36%	1***	1**	42%	1***	1
Fair/poor	21%	0.61 (0.50, 0.74)	0.74 (0.61, 0.90)	26%	0.65 (0.53, 0.79)	0.83 (0.68, 1.02)
Long-term illness	No	36%	1***	1	42%	1*	1
Yes	24%	0.73 (0.61, 0.87)	0.93 (0.78, 1.12)	28%	0.77 (0.63, 0.95)	0.97 (0.79, 1.20)
Time walking or cycling in past week (minutes)	None	17%	1***	1***	19%	1***	1***
1–149	30%	1.61 (1.23, 2.10)	1.41 (1.08, 1.85)	35%	1.65 (1.20, 2.26)	1.47 (1.10, 1.96)
150–299	30%	1.65 (1.27, 2.14)	1.41 (1.10, 1.81)	38%	1.81 (1.35, 2.43)	1.52 (1.16, 1.99)
300–449	38%	2.08 (1.63, 2.66)	1.69 (1.32, 2.16)	46%	2.22 (1.67, 2.95)	1.83 (1.41, 2.38)
≥ 450	45%	2.32 (1.76, 3.05)	1.93 (1.47, 2.53)	51%	2.37 (1.71, 3.27)	2.09 (1.55, 2.81)

*p < 0.05, **p < 0.01, ***p < 0.001 for heterogeneity. CI = confidence interval, km = kilometers; RR = relative risk. Minimally-adjusted analyses adjusted for site, sex and age only, multivariable analyses adjusted for all variables in column. Intermediate multivariable models (e.g. including only demographic and socio-economic characteristics) available on request from the authors. Results based on 1849 British adults participating in 2010 and 2011, and 1510 participating in 2010 and 2012.
