# Supplementary material for: Who uses new walking and cycling infrastructure and how? Longitudinal results from the UK iConnect study
Source: Prev Med. 2013 Nov;57(5):518–24. doi: 10.1016/j.ypmed.2013.07.007 (PMC3807875; doi:10.1016/j.ypmed.2013.07.007)
Supplement: Supplementary file 1 — Supplementary material. [file mmc1.doc]

**Supplementary material, part : Representativeness of the study sample**

Table 1: Comparison of study sample with general population

| **Domain** | **Baseline characteristics** | **Level** | **Study sample, weighted by age & sex (%)** | | **General population** | **Comparison population** |
| --- | --- | --- | --- | --- | --- | --- |
|  |  |  | **One year (N=1849)** | **Two year (N=1510)** | **(%)** |  |
| Demo- | Sex | Female | 51 | 51 | 51 | a Local: Office |
| graphic |  | Male | 49 | 49 | 49 | for National |
|  | Age (years) | 18-29 | 26 | 25 | 26 | Statistics 2010 |
|  |  | 30-49 | 35 | 35 | 35 |  |
|  |  | 50-64 | 22 | 22 | 22 |  |
|  |  | 65+ | 17 | 18 | 17 |  |
|  | Ethnicity | White | 94 | 94 | 94 | b Local: Census |
|  |  | Non-White | 6 | 6 | 6 | 2001 |
|  | Any child | No | 78 | 77 | 60 |  |
|  | under 16 | Yes | 22 | 23 | 40 |  |
|  | Urban/rural | Urban | 96 | 96 | 94 |  |
|  | status | Rural | 4 | 5 | 6 |  |
| Socio- | Highest | Degree | 44 | 46 | 26 |  |
| economic | educational | A-level | 20 | 21 | 11 |  |
|  | qualification | GCSE | 16 | 15 | 16 |  |
|  |  | None or other | 20 | 18 | 46 |  |
|  | Tenure | Home owner | 78 | 79 | 70 |  |
|  |  | Renting | 22 | 21 | 31 |  |
|  | Employment | Employed | 64 | 62 | 64 |  |
|  | status | Unemployed | 2 | 2 | 3 |  |
|  |  | Student | 7 | 8 | 6 |  |
|  |  | Other economically inactive | 27 | 28 | 27 |  |
| Health | Weight status | Normal/underweight | 57 | 57 | 39 | c National: Health |
|  |  | Overweight | 32 | 32 | 38 | survey for |
|  |  | Obese | 11 | 11 | 23 | England 2009 |
|  | General | Excellent/good | 79 | 77 | 63 | b Local: Census |
|  | health | Fair/poor | 21 | 23 | 37 | 2001 |
|  | Long-term | No | 82 | 83 | 79 |  |
|  | limiting illness | Yes | 18 | 17 | 21 |  |
| Travel | Cars per adult | No cars | 15 | 15 | 20 |  |
|  | in household | <1 car per adult | 39 | 39 | 35 |  |
|  |  | ≥1 cars per adult | 46 | 46 | 44 |  |
|  | Main mode to | Car | 70 | 72 | 73 |  |
|  | work (mode | Public transport | 12 | 12 | 10 |  |
|  | involving the | Walk | 10 | 9 | 13 |  |
|  | greatest distance) | Cycle | 9 | 7 | 4 |  |
|  | Percentage travel | Car | 75 | 77 | 78 | d National: |
|  | distance covered | Bus or train | 17 | 15 | 14 | National Travel |
|  | by different modes | Walk | 4 | 4 | 3 | Survey, 2010 |
|  |  | Cycle | 2 | 2 | 1 |  |
|  |  | Other modes | 2 | 2 | 4 |  |

^a^ ONS mid-2010 population estimates (Office for National Statistics 2011), percentages calculated by authors. We included all adult residents (aged ≥16 years) living in the three local authorities from which we drew our study samples, giving equal weighting to each local authority.

^b^ Census 2001 5% sample in Small Area Microdata (Office for National Statistics 2004), percentages calculated by authors. We included all adult residents (aged >20 years) living in private households in the three local authorities from which we drew our study samples, giving equal weighting to each local authority. To ensure comparability, we also restricted our study sample to those ages 20 or more (97% of sample) when making comparisons with the census data.

^c^ Health Survey for England 2009, adult sample (NHS Information Centre 2010)

^d^ National Travel Survey 2010 (Department for Transport 2009).

**Table 2: Predictors of remaining in the study sample from baseline (N=3516) to one-year follow up (N=1849) and two-year follow up (N=1510)**

| **Baseline** | **Level** | **N at** | **One-year follow-up** | | **Two-year follow-up** | |
| --- | --- | --- | --- | --- | --- | --- |
| **characteristics** |  | **baseline** | **% follow-ed up** | **Adjusted RR (95%CI)** † | **% follow-ed up** | **Adjusted RR (95%CI)** † |
| Site | Southampton | 1127 | 46% | 1 | 38% | 1 |
|  | Cardiff | 1124 | 53% | 0.99 (0.91, 1.08) | 43% | 0.98 (0.89, 1.08) |
|  | Kenilworth | 1265 | 58% | 1.01 (0.93, 1.09) | 47% | 0.95 (0.86, 1.05) |
| Proximity of | ≥4 | 742 | 54% | [not | 43% | [not |
| home to | 3-3.99 | 680 | 57% | included] | 48% | included] |
| planned core | 2-2.99 | 958 | 55% |  | 47% |  |
| Connect2 (km) | 1-1.99 | 866 | 48% |  | 38% |  |
|  | <1 | 270 | 43% |  | 34% |  |
| Proximity of | ≥4 | 348 | 55% | 1 | 46% | 1 |
| home to | 3-3.99 | 299 | 60% | 1.10 (0.96, 1.26) | 45% | 0.97 (0.82, 1.14) |
| planned greater | 2-2.99 | 638 | 53% | 1.04 (0.92, 1.18) | 43% | 1.00 (0.86, 1.16) |
| Connect2 (km) | 1-1.99 | 1187 | 54% | 1.05 (0.94, 1.17) | 44% | 1.01 (0.89, 1.15) |
|  | <1 | 1044 | 48% | 0.99 (0.88, 1.11) | 40% | 0.99 (0.87, 1.13) |
| Sex | Female | 1924 | 52% | 1 | 45% | 1 |
|  | Male | 1570 | 53% | 0.97 (0.92, 1.03) | 42% | 0.87 (0.81, 0.94) |
| Age (change per decade) | Linear term | - | - | 1.63 (1.42, 1.87)*** | - | 2.55 (2.13, 3.06)*** |
|  | Quadratic term | - | - | 0.97 (0.95, 0.98)*** | - | 0.93 (0.92, 0.95)*** |
| Ethnicity | White | 3268 | 54% | 1 | 45% | 1 |
|  | Non-White | 185 | 34% | 0.79 (0.65, 0.97) | 24% | 0.69 (0.53, 0.89) |
| Any child | No | 2771 | 55% | 1 | 46% | 1 |
| under 16 | Yes | 708 | 42% | 0.93 (0.84, 1.03) | 33% | 0.96 (0.85, 1.09) |
| Education | Tertiary | 1377 | 52% | 1 | 43% | 1* |
|  | Secondary school | 1254 | 50% | 0.95 (0.88, 1.03) | 39% | 0.87 (0.79, 0.95) |
|  | None or other | 814 | 61% | 0.99 (0.91, 1.08) | 52% | 0.92 (0.83, 1.02) |
| Annual | >£40,000 | 1084 | 51% | 1 | 41% | 1 |
| household | £20,001-40,000 | 981 | 52% | 0.97 (0.89, 1.05) | 47% | 1.07 (0.97, 1.19) |
| income | ≤£20,000 | 962 | 55% | 0.97 (0.88, 1.07) | 50% | 1.03 (0.91, 1.16) |
| Employment | Working | 1888 | 50% | 1** | 39% | 1 |
| status | Student | 223 | 22% | 0.75 (0.56, 1.01) | 11% | 0.75 (0.50, 1.15) |
|  | Retired | 999 | 71% | 1.10 (1.01, 1.21) | 61% | 1.09 (0.98, 1.21) |
|  | Other | 369 | 41% | 0.87 (0.76, 0.99) | 36% | 0.95 (0.82, 1.10) |
| Any car | No | 534 | 46% | 1* | 40% | 1 |
| in household | Yes | 2976 | 54% | 1.12 (1.01, 1.24) | 43% | 1.03 (0.92, 1.16) |
| Any adult bicycle | No | 1388 | 55% | 1 | 45% | 1 |
| in household | Yes | 1894 | 51% | 0.99 (0.92, 1.07) | 41% | 1.03 (0.95, 1.12) |
| Weight status | Normal | 1694 | 52% | 1 | 42% | 1 |
|  | Overweight | 1151 | 55% | 0.96 (0.89, 1.03) | 46% | 0.99 (0.91, 1.07) |
|  | Obese | 477 | 51% | 0.90 (0.82, 1.00) | 42% | 0.90 (0.80, 1.01) |
| General | Excellent/good | 2684 | 54% | 1 | 44% | 1 |
| health | Fair/poor | 782 | 50% | 0.96 (0.88, 1.04) | 41% | 0.98 (0.88, 1.08) |
| Long-term | No | 2,566 | 51% | 1 | 41% | 1 |
| illness | Yes | 742 | 59% | 1.01 (0.93, 1.09) | 50% | 1.01 (0.92, 1.12) |
| Walking or | None | 575 | 51% | 1 | 41% | 1 |
| cycling for | 1-149 | 930 | 52% | 1.07 (0.97, 1.18) | 41% | 1.10 (0.97, 1.24) |
| transport or | 150-299 | 774 | 54% | 1.10 (0.99, 1.22) | 46% | 1.20 (1.06, 1.36) |
| recreation in past | 300-449 | 512 | 53% | 1.11 (0.99, 1.24) | 43% | 1.14 (0.99, 1.31) |
| week (min) ‡ | ≥450 | 725 | 54% | 1.12 (1.01, 1.24) | 43% | 1.13 (1.00, 1.29) |

*p<0.05, **p<0.01, ***p<0.001 for heterogeneity. CI=confidence interval, RR=relative risk. †Rate ratio for inclusion at follow-up, adjusted for all variables in column. Age was a very strong predictor of retention (see next page) and so was entered as a linear plus a quadratic term to minimise residual confounding. ‡ also no evidence when split into four separate behaviours of walking or cycling for transport or recreation.

As shown in Figure 1, age showed a very strong association with the probability of participation in the one and two-year follow-ups. Table 2 therefore includes adjustment for age as linear plus quadratic terms in order to minimise residual confounding.

Figure 1: Proportion of the study population followed up at one- and two-years, by age at baseline


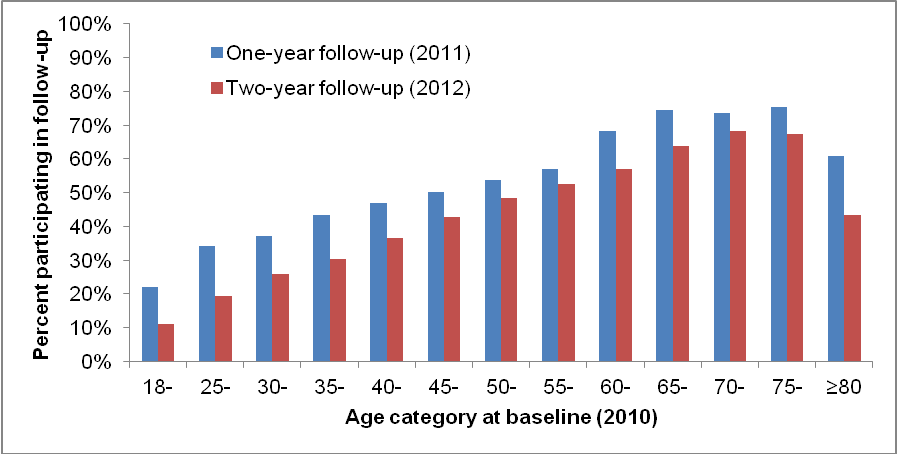


**References**

Department for Transport (2009). National Travel Survey: Why People Travel;. [<http://assets.dft.gov.uk/statistics/releases/national-travel-survey-2010/nts2010-03.pdf>, accessed 14/11/2011].

NHS Information Centre (2010). Health Survey for England 2009: Trend tables. [<http://www.ic.nhs.uk/statistics-and-data-collections/health-and-lifestyles-related-surveys/health-survey-for-england/health-survey-for-england--2009-trend-tables>, accessed 14/11/2011].

Office for National Statistics (2004). Census 2001 Small Area Microfile, Available via <http://www.ons.gov.uk/ons/about-ons/who-we-are/services/unpublished-data/census-data/sam/index.html>.

Office for National Statistics (2011). Population Estimates for UK, England and Wales, Scotland and Northern Ireland: Mid-2010 Population Estimates. London, Office for National Statistics.

**Supplementary material, part : Maps of Connect2 intervention**

Figure 2: Locations of case study sites

Reproduced with the permission of Sustrans.

Figure 3: ‘Core’ and ‘greater’ Connect2 projects at the Kenilworth study site


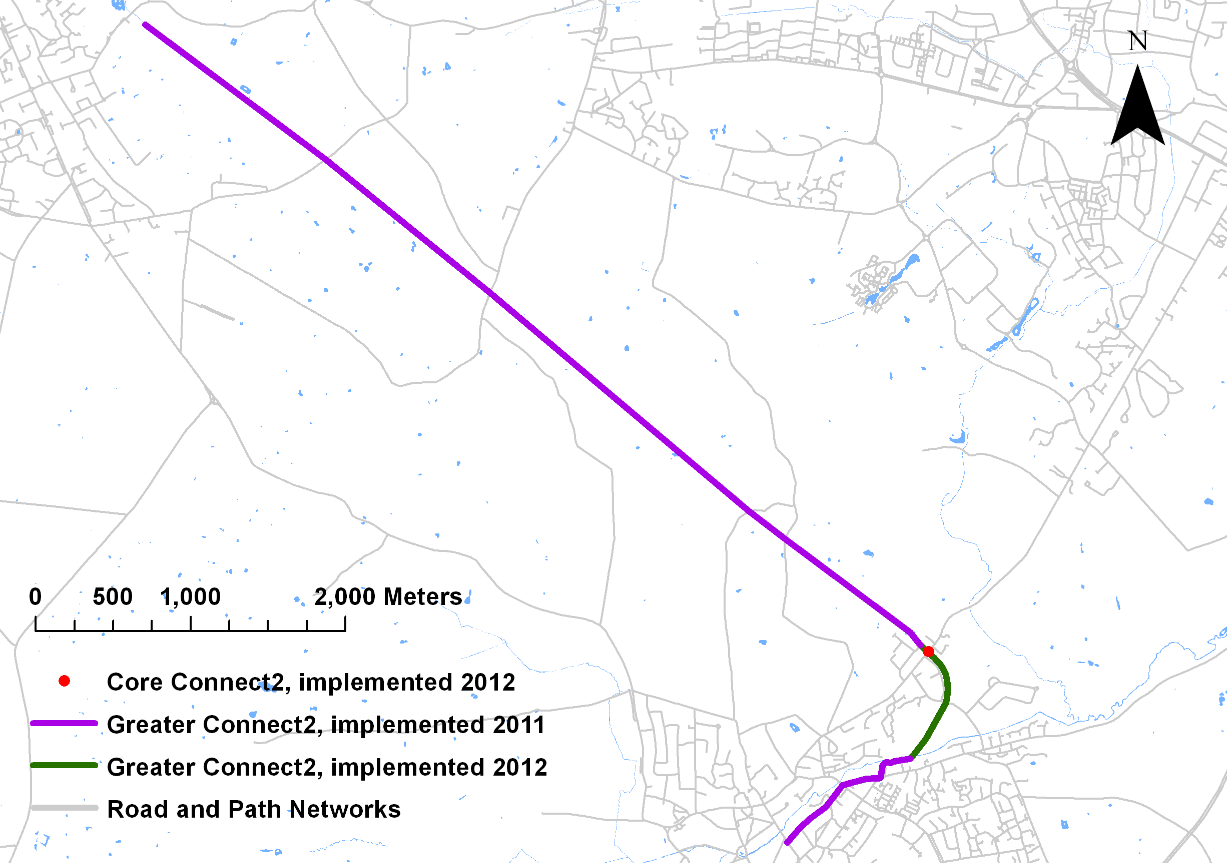


Contains Ordnance Survey data © Crown copyright and database right 2011.

Figure 4: ‘Core’ and ‘greater’ Connect2 projects at the Southampton study site


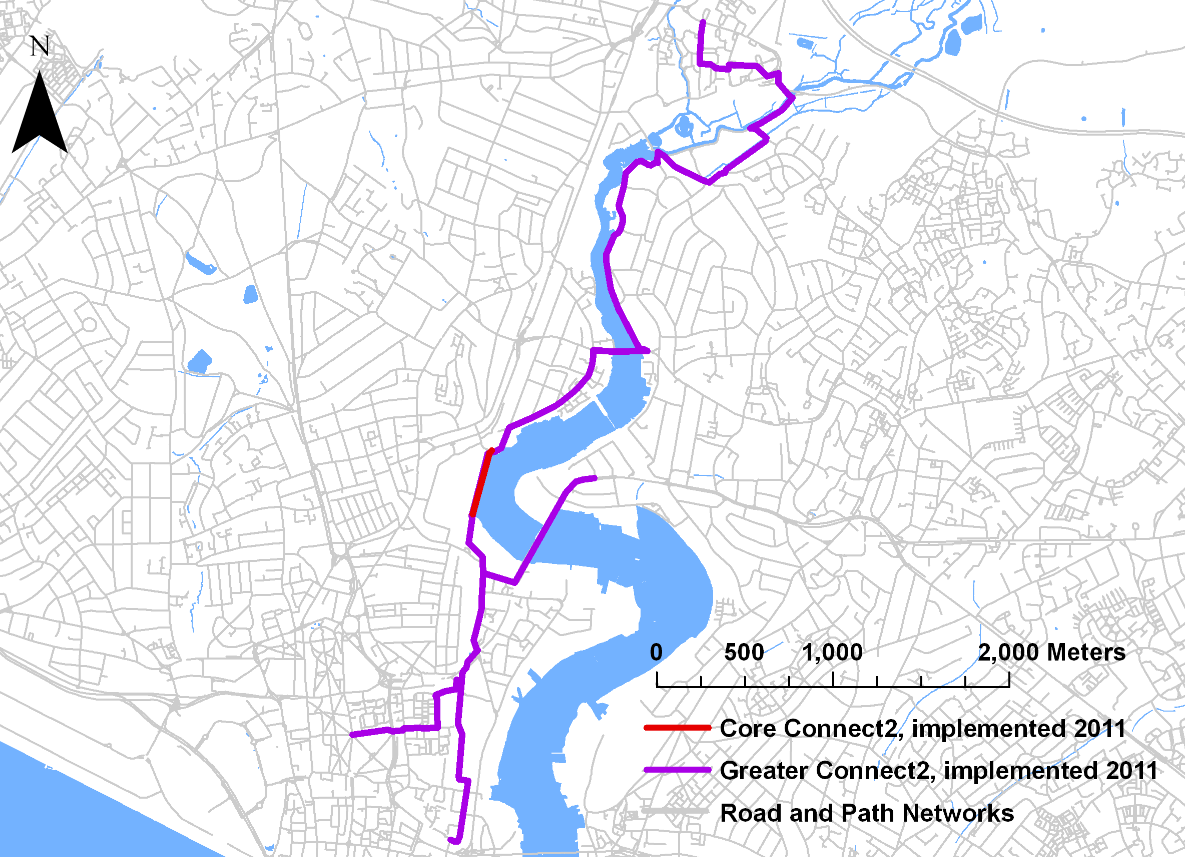


Contains Ordnance Survey data © Crown copyright and database right 2011.

**Supplementary material, part : Additional analyses relating to use of Connect2**

**Table 3: Number of individuals reporting using Connect2 for different purposes at one- and two-year follow-up and across years**

|  |  | **One-year sample (N=1826)** | **Two-year sample (N=1490)** | **One+two year sample (N=1235)** | | | |
| --- | --- | --- | --- | --- | --- | --- | --- |
|  |  | **Used at one year** | **Used at two years** | **Did not use at either follow-up** | **Used at one year only** | **Used at two years only** | **Used in both years** |
| Walking | Any transport | 205 | 179 | 1036 | 58 | 63 | 78 |
|  | Social/leisure | 152 | 126 | 1087 | 50 | 46 | 52 |
|  | Shopping/personal | 103 | 85 | 1132 | 33 | 37 | 33 |
|  | To work | 21 | 19 | 1214 | 8 | 8 | 5 |
|  | For education | 10 | 8 | 1227 | 4 | 2 | 2 |
|  | For business | 9 | 14 | 1222 | 2 | 7 | 4 |
|  | Recreation | 492 | 481 | 750 | 77 | 138 | 270 |
|  | Any walking | 537 | 515 | 726 | 81 | 135 | 293 |
| Cycling | Any transport | 97 | 103 | 1136 | 18 | 37 | 44 |
|  | Social/leisure | 66 | 71 | 1160 | 23 | 32 | 20 |
|  | Shopping/personal | 41 | 35 | 1194 | 13 | 17 | 11 |
|  | To work | 27 | 29 | 1208 | 5 | 10 | 12 |
|  | For education | 4 | 7 | 1228 | 0 | 3 | 4 |
|  | For business | 8 | 14 | 1221 | 4 | 7 | 3 |
|  | Recreation | 214 | 220 | 1024 | 30 | 69 | 112 |
|  | Any cycling | 232 | 242 | 1008 | 31 | 72 | 124 |
| Use Connect2 for any purpose | | 586 | 563 | 691 | 74 | 141 | 329 |

Analyses exclude 23 participants (1.2%) in the one-year sample who did not answer this question; 20 participants (1.3%) in the two-year sample who did not answer this question; and 22 participants (1.8%) in the one+two year sample who did not answer these questions.

**Figure 5: Effect of proximity of Connect2 and baseline walking and cycling on different types of Connect2 use at one-year follow-up**

CR=cycling for recreation; CT=cycling for transport; CI=confidence interval, km=kilometers; min= minutes; WR=walking for recreation; WT=walking for transport. Results very similar at two-year follow-up, see main text.

Tables summarising the predictors of awareness of the Connect2 projects at one- and two-year follow-up are available from the authors on request.

**Table 4: Predictors of using Connect2 for different types of behaviours at one-year follow-up**

| **Baseline** | **Level** | **Using Connect2 for WT** | | **Using Connect2 for WR** | | **Using Connect2 for CT** | | **Using Connect2 for CR** | |
| --- | --- | --- | --- | --- | --- | --- | --- | --- | --- |
| **characteristic** |  | **%** | **Multivariable**  **RR (95%CI)** | **%** | **Multivariable**  **RR (95%CI)** | **%** | **Multivariable**  **RR (95%CI)** | **%** | **Multivariable**  **RR (95%CI)** |
| Site | Southampton | 8% | 1*** | 15% | 1*** | 4% | 1** | 6% | 1*** |
|  | Cardiff | 20% | 2.15 (1.40, 3.32) | 41% | 2.29 (1.68, 3.11) | 10% | 2.18 (1.30, 3.67) | 18% | 2.86 (1.80, 4.56) |
|  | Kenilworth | 7% | 1.15 (0.71, 1.85) | 24% | 1.71 (1.23, 2.37) | 3% | 1.00 (0.51, 1.96) | 12% | 2.35 (1.49, 3.70) |
| Proximity of | ≥4 | 2% | 1*** | 9% | 1*** | 0% | [small cell†] | 3% | 1*** |
| home to greater | 3-3.99 | 9% | 3.08 (0.92, 10.30) | 20% | 1.84 (1.04, 3.27) | 7% | 1** | 9% | 2.90 (0.97, 8.71) |
| Connect2 (km) | 2-2.99 | 6% | 1.85 (0.63, 5.41) | 20% | 2.29 (1.27, 4.15) | 2% | [small cell†] | 9% | 3.01 (1.07, 8.47) |
|  | 1-1.99 | 8% | 2.92 (1.07, 8.02) | 24% | 2.91 (1.62, 5.24) | 4% | 1.74 (0.84, 3.59) | 13% | 4.48 (1.64, 12.22) |
|  | <1 | 20% | 6.05 (2.26, 16.17) | 40% | 4.53 (2.59, 7.91) | 10% | 3.30 (1.72, 6.33) | 16% | 5.36 (1.97, 14.55) |
| Sex | Female | 10% | 1 | 27% | 1 | 4% | 1 | 10% | 1* |
|  | Male | 13% | 1.24 (0.95, 1.61) | 27% | 0.98 (0.86, 1.12) | 7% | 1.47 (0.90, 2.40) | 15% | 1.34 (1.02, 1.76) |
| Age (years) | 18-34 | 20% | 1** | 27% | 1 | 8% | 1 | 13% | 1 |
|  | 35-49 | 13% | 0.64 (0.45, 0.90) | 31% | 1.02 (0.80, 1.30) | 10% | 0.95 (0.46, 1.98) | 23% | 1.24 (0.90, 1.70) |
|  | 50-64 | 12% | 0.63 (0.45, 0.89) | 31% | 1.10 (0.83, 1.45) | 4% | 0.73 (0.39, 1.36) | 10% | 0.85 (0.61, 1.20) |
|  | >65 | 7% | 0.45 (0.28, 0.72) | 21% | 0.86 (0.60, 1.24) | 3% | 1.45 (0.47, 4.52) | 7% | 0.91 (0.52, 1.57) |
| Ethnicity | White | 11% | 1 | 28% | 1 | 5% | 1 | 12% | 1 |
|  | Non-White | 16% | 1.31 (0.76, 2.25) | 14% | 0.62 (0.35, 1.10) | 5% | 0.69 (0.19, 2.47) | 13% | 1.11 (0.63, 1.95) |
| Any child | No | 11% | 1 | 26% | 1 | 4% | 1 | 9% | 1 |
| under 16 | Yes | 15% | 1.05 (0.71, 1.55) | 33% | 1.05 (0.82, 1.33) | 12% | 1.30 (0.75, 2.24) | 26% | 1.32 (0.99, 1.76) |
| Education | Tertiary | 13% | 1 | 35% | 1 | 9% | 1 | 18% | 1 |
|  | Secondary | 12% | 1.19 (0.89, 1.60) | 24% | 0.91 (0.78, 1.06) | 4% | 0.72 (0.44, 1.19) | 9% | 0.73 (0.54, 0.99) |
|  | None or other | 8% | 1.12 (0.76, 1.66) | 20% | 0.96 (0.81, 1.15) | 3% | 0.78 (0.45, 1.36) | 7% | 0.90 (0.67, 1.21) |
| Annual | >£40,000 | 13% | 1 | 35% | 1 | 8% | 1 | 20% | 1 |
| household | £20-40,000 | 11% | 0.87 (0.61, 1.25) | 28% | 0.91 (0.74, 1.12) | 5% | 1.00 (0.58, 1.73) | 10% | 0.80 (0.58, 1.09) |
| income | ≤£20,000 | 10% | 1.00 (0.63, 1.58) | 19% | 0.76 (0.58, 1.01) | 3% | 0.98 (0.50, 1.90) | 7% | 0.82 (0.59, 1.15) |
| Employment | Working | 14% | 1 | 30% | 1* | 7% | 1 | 16% | 1 |
| status | Student | 8% | 0.39 (0.15, 1.02) | 8% | 0.35 (0.15, 0.82) | 4% | 0.57 (0.13, 2.47) | 6% | 0.48 (0.18, 1.33) |
|  | Retired | 9% | 1.12 (0.74, 1.67) | 25% | 1.22 (0.97, 1.55) | 3% | 0.62 (0.22, 1.72) | 7% | 1.09 (0.67, 1.77) |
|  | Other | 13% | 0.92 (0.58, 1.48) | 22% | 0.86 (0.65, 1.13) | 5% | 0.96 (0.47, 1.96) | 9% | 0.76 (0.47, 1.23) |
| Any car | No | 13% | 1 | 16% | 1 | 5% | 1 | 8% | 1 |
| in household | Yes | 11% | 0.69 (0.47, 1.02) | 29% | 1.19 (0.90, 1.59) | 6% | 0.69 (0.37, 1.28) | 13% | 0.67 (0.40, 1.15) |
| Any adult bike | No | 7% | 1*** | 19% | 1*** | 1% | 1** | 2% | 1*** |
| in household | Yes | 15% | 1.83 (1.36, 2.46) | 34% | 1.41 (1.15, 1.72) | 9% | 4.69 (1.78, 12.35) | 20% | 5.40 (3.27, 8.93) |
| Weight status | Normal | 12% | 1 | 28% | 1 | 6% | 1 | 13% | 1 |
|  | Overweight | 11% | 1.02 (0.80, 1.29) | 28% | 1.09 (0.92, 1.30) | 5% | 1.08 (0.76, 1.52) | 12% | 1.14 (0.88, 1.48) |
|  | Obese | 9% | 0.91 (0.59, 1.40) | 21% | 0.98 (0.77, 1.25) | 5% | 1.46 (0.78, 2.73) | 9% | 1.37 (0.86, 2.20) |
| General | Excellent/good | 12% | 1 | 30% | 1*** | 6% | 1 | 14% | 1 |
| health | Fair/poor | 8% | 0.71 (0.48, 1.04) | 15% | 0.68 (0.55, 0.84) | 3% | 0.71 (0.35, 1.46) | 5% | 0.61 (0.40, 0.93) |
| Long-term | No | 13% | 1 | 30% | 1 | 6% | 1 | 14% | 1* |
| illness | Yes | 8% | 1.07 (0.75, 1.51) | 18% | 0.91 (0.75, 1.10) | 3% | 0.94 (0.49, 1.82) | 6% | 0.86 (0.57, 1.29) |
| Walking | None | 5% | 1** | 20% | 1** | 3% | 1 | 10% | 1 |
| for transport in | 1-149 | 14% | 1.95 (1.33, 2.85) | 32% | 1.33 (1.13, 1.56) | 6% | 1.32 (0.83, 2.10) | 13% | 0.92 (0.66, 1.29) |
| past week (min) | 150-299 | 11% | 1.74 (1.02, 2.99) | 29% | 1.43 (1.14, 1.79) | 5% | 1.45 (0.74, 2.85) | 12% | 1.15 (0.79, 1.67) |
|  | 300-449 | 17% | 2.67 (1.58, 4.51) | 29% | 1.24 (0.91, 1.68) | 9% | 2.05 (1.05, 3.99) | 11% | 0.83 (0.53, 1.29) |
|  | ≥450 | 20% | 2.61 (1.53, 4.45) | 31% | 1.29 (0.95, 1.75) | 9% | 1.73 (0.76, 3.93) | 15% | 1.37 (0.82, 2.31) |
| Walking | None | 8% | 1 | 17% | 1*** | 4% | 1 | 8% | 1* |
| for recreation in | 1-149 | 12% | 1.14 (0.85, 1.52) | 29% | 1.28 (1.06, 1.54) | 5% | 0.82 (0.54, 1.26) | 12% | 1.11 (0.76, 1.63) |
| past week (min) | 150-299 | 14% | 1.34 (0.89, 2.02) | 34% | 1.48 (1.21, 1.82) | 7% | 1.20 (0.66, 2.17) | 16% | 1.45 (0.98, 2.16) |
|  | 300-449 | 13% | 1.25 (0.78, 2.02) | 39% | 1.72 (1.34, 2.22) | 8% | 1.34 (0.80, 2.25) | 17% | 1.67 (1.13, 2.48) |
|  | ≥450 | 16% | 1.61 (0.96, 2.70) | 43% | 2.26 (1.72, 2.99) | 5% | 1.16 (0.42, 3.19) | 13% | 1.31 (0.76, 2.26) |
| Cycling | None | 10% | 1 | 25% | 1 | 3% | 1*** | 9% | 1 |
| for transport in | 1-149 | 18% | 0.96 (0.57, 1.61) | 40% | 1.14 (0.89, 1.46) | 15% | 2.13 (1.21, 3.72) | 33% | 1.47 (1.08, 2.01) |
| past week (min) | 150-299 | 18% | 0.76 (0.45, 1.29) | 37% | 0.88 (0.61, 1.26) | 32% | 2.78 (1.60, 4.82) | 34% | 0.95 (0.56, 1.63) |
|  | ≥300 | 23% | 1.21 (0.55, 2.64) | 50% | 1.36 (0.86, 2.13) | 41% | 4.68 (2.22, 9.85) | 50% | 1.33 (0.87, 2.04) |
| Cycling | None | 10% | 1 | 25% | 1 | 4% | 1 | 8% | 1*** |
| for recreation in | 1-149 | 20% | 1.36 (0.79, 2.34) | 43% | 1.25 (1.01, 1.54) | 16% | 1.41 (0.78, 2.54) | 36% | 1.81 (1.37, 2.38) |
| past week (min) | 150-299 | 20% | 1.32 (0.75, 2.31) | 44% | 1.22 (0.84, 1.78) | 20% | 1.36 (0.73, 2.54) | 50% | 2.50 (1.75, 3.56) |
|  | ≥300 | 16% | 0.87 (0.28, 2.74) | 45% | 1.05 (0.67, 1.63) | 23% | 1.44 (0.53, 3.88) | 48% | 2.45 (1.56, 3.83) |

*p<0.05, **p<0.01, ***p<0.001 for heterogeneity. WT=walking for transport, WR=walking for recreation, CT=cycling for transport, CR=cycling for recreation, CI=confidence interval, RR=relative risk. Multivariable analyses adjusted for all variables in the column. † indicates levels combined with the adjacent category because of small cell sizes (<5 Connect2 users). †Cells merged with reference group because of small cell sizes (<5 Connect2 users).

**Table 5: Predictors of using Connect2 for different types of behaviours at two-year follow-up**

| **Baseline** | **Level** | **Using Connect2 for WT** | | **Using Connect2 for WR** | | **Using Connect2 for CT** | | **Using Connect2 for CR** | |
| --- | --- | --- | --- | --- | --- | --- | --- | --- | --- |
| **characteristic** |  | **%** | **Multivariable**  **RR (95%CI)** | **%** | **Multivariable**  **RR (95%CI)** | **%** | **Multivariable**  **RR (95%CI)** | **%** | **Multivariable**  **RR (95%CI)** |
| Site | Southampton | 9% | 1*** | 16% | 1*** | 6% | 1*** | 8% | 1*** |
|  | Cardiff | 21% | 2.25 (1.48, 3.43) | 47% | 2.59 (2.03, 3.31) | 12% | 2.13 (1.37, 3.32) | 21% | 2.62 (1.88, 3.63) |
|  | Kenilworth | 7% | 1.00 (0.61, 1.62) | 33% | 2.11 (1.65, 2.70) | 4% | 0.83 (0.46, 1.48) | 15% | 2.08 (1.45, 2.97) |
| Proximity of | ≥4 | 5% | 1*** | 9% | 1*** | 3% | 1** | 4% | 1*** |
| home to greater | 3-3.99 | 8% | 1.11 (0.34, 3.62) | 26% | 2.30 (1.24, 4.27) | 5% | 0.89 (0.19, 4.11) | 9% | 2.10 (0.77, 5.72) |
| Connect2 (km) | 2-2.99 | 6% | 0.75 (0.27, 2.09) | 22% | 2.56 (1.48, 4.44) | 3% | 0.71 (0.20, 2.50) | 11% | 2.98 (1.22, 7.29) |
|  | 1-1.99 | 9% | 1.43 (0.58, 3.52) | 31% | 3.83 (2.24, 6.55) | 6% | 1.35 (0.43, 4.25) | 16% | 4.94 (2.14, 11.39) |
|  | <1 | 21% | 3.04 (1.26, 7.34) | 46% | 5.07 (2.95, 8.70) | 11% | 2.29 (0.77, 6.78) | 19% | 5.47 (2.35, 12.72) |
| Sex | Female | 12% | 1 | 33% | 1 | 5% | 1 | 12% | 1** |
|  | Male | 13% | 1.01 (0.78, 1.32) | 32% | 0.94 (0.82, 1.07) | 9% | 1.41 (0.96, 2.07) | 19% | 1.38 (1.09, 1.76) |
| Age (years) | 18-34 | 17% | 1 | 31% | 1* | 10% | 1 | 19% | 1 |
|  | 35-49 | 13% | 0.76 (0.45, 1.28) | 37% | 0.97 (0.75, 1.25) | 13% | 0.99 (0.58, 1.69) | 26% | 0.81 (0.56, 1.18) |
|  | 50-64 | 15% | 0.93 (0.59, 1.48) | 39% | 1.13 (0.88, 1.44) | 6% | 0.77 (0.43, 1.35) | 15% | 0.74 (0.51, 1.09) |
|  | >65 | 8% | 0.58 (0.33, 1.02) | 24% | 0.84 (0.62, 1.13) | 3% | 0.62 (0.26, 1.46) | 7% | 0.61 (0.38, 0.99) |
| Ethnicity | White | 13% | 1 | 33% | 1 | 7% | 1 | 15% | 1 |
|  | Non-White | 2% | 0.20 (0.03, 1.43) | 18% | 0.85 (0.50, 1.44) | 2% | 0.30 (0.03, 2.67) | 12% | 1.03 (0.44, 2.43) |
| Any child | No | 12% | 1 | 31% | 1 | 5% | 1 | 12% | 1 |
| under 16 | Yes | 14% | 1.12 (0.69, 1.83) | 39% | 1.15 (0.92, 1.42) | 16% | 1.49 (0.96, 2.33) | 31% | 1.32 (1.01, 1.72) |
| Education | Tertiary | 14% | 1 | 42% | 1 | 11% | 1 | 24% | 1* |
|  | Secondary | 12% | 0.93 (0.66, 1.31) | 30% | 0.87 (0.73, 1.04) | 5% | 0.57 (0.34, 0.93) | 11% | 0.68 (0.50, 0.92) |
|  | None or other | 10% | 0.96 (0.68, 1.36) | 23% | 0.89 (0.71, 1.11) | 4% | 0.72 (0.40, 1.30) | 8% | 0.79 (0.53, 1.16) |
| Annual | >£40,000 | 13% | 1 | 44% | 1 | 10% | 1 | 25% | 1 |
| household | £20-40,000 | 12% | 1.00 (0.69, 1.45) | 34% | 0.93 (0.80, 1.09) | 6% | 1.16 (0.75, 1.79) | 14% | 0.93 (0.73, 1.18) |
| income | ≤£20,000 | 12% | 1.08 (0.68, 1.74) | 21% | 0.77 (0.60, 0.98) | 5% | 1.38 (0.82, 2.34) | 7% | 0.77 (0.53, 1.11) |
| Employment | Working | 14% | 1 | 38% | 1** | 10% | 1 | 20% | 1 |
| status | Student | 4% | 0.26 (0.04, 1.86) | 8% | 0.27 (0.09, 0.85) | 0% | [small cell†] | 4% | 0.21 (0.03, 1.29) |
|  | Retired | 10% | 1.05 (0.73, 1.52) | 29% | 1.14 (0.95, 1.38) | 4% | 0.91 (0.47, 1.74) | 9% | 1.03 (0.73, 1.44) |
|  | Other | 16% | 1.02 (0.63, 1.65) | 23% | 0.73 (0.54, 0.99) | 8% | 1.15 (0.61, 2.18) | 16% | 1.19 (0.77, 1.83) |
| Any car | No | 18% | 1* | 20% | 1 | 7% |  | 7% | 1 |
| in household | Yes | 11% | 0.61 (0.42, 0.90) | 35% | 1.04 (0.80, 1.35) | 7% | 0.63 (0.34, 1.15) | 16% | 0.87 (0.49, 1.54) |
| Any adult bike | No | 10% | 1 | 23% | 1*** | 2% | 1** | 3% | 1*** |
| in household | Yes | 14% | 1.32 (0.95, 1.83) | 41% | 1.38 (1.15, 1.64) | 11% | 2.34 (1.31, 4.17) | 25% | 4.69 (2.95, 7.46) |
| Weight status | Normal | 12% | 1 | 34% | 1 | 9% | 1 | 17% | 1 |
|  | Overweight | 13% | 1.15 (0.81, 1.63) | 33% | 1.06 (0.89, 1.26) | 6% | 0.99 (0.63, 1.56) | 15% | 1.02 (0.80, 1.30) |
|  | Obese | 11% | 0.96 (0.60, 1.53) | 25% | 0.98 (0.73, 1.31) | 4% | 0.75 (0.36, 1.57) | 8% | 0.90 (0.53, 1.52) |
| General | Excellent/good | 13% | 1 | 36% | 1 | 8% | 1 | 17% | 1 |
| health | Fair/poor | 11% | 0.93 (0.63, 1.39) | 20% | 0.85 (0.67, 1.07) | 5% | 1.08 (0.62, 1.88) | 8% | 0.87 (0.54, 1.40) |
| Long-term | No | 13% | 1 | 36% | 1 | 8% | 1 | 18% | 1 |
| illness | Yes | 10% | 0.91 (0.59, 1.40) | 23% | 0.96 (0.76, 1.22) | 4% | 1.04 (0.53, 2.04) | 8% | 1.05 (0.70, 1.58) |
| Walking | None | 8% | 1** | 28% | 1 | 5% | 1 | 14% | 1 |
| for transport in | 1-149 | 13% | 1.23 (0.86, 1.76) | 37% | 1.10 (0.94, 1.30) | 9% | 0.93 (0.60, 1.44) | 17% | 0.91 (0.72, 1.14) |
| past week (min) | 150-299 | 15% | 1.69 (1.10, 2.60) | 28% | 0.96 (0.76, 1.21) | 5% | 0.82 (0.46, 1.46) | 10% | 0.77 (0.52, 1.13) |
|  | 300-449 | 14% | 1.57 (0.85, 2.90) | 33% | 1.05 (0.80, 1.39) | 7% | 0.74 (0.29, 1.89) | 15% | 0.88 (0.57, 1.37) |
|  | ≥450 | 26% | 2.60 (1.50, 4.49) | 43% | 1.43 (1.07, 1.90) | 11% | 1.47 (0.70, 3.09) | 21% | 1.60 (1.08, 2.38) |
| Walking | None | 10% | 1 | 20% | 1*** | 5% | 1 | 10% | 1 |
| for recreation in | 1-149 | 13% | 1.12 (0.79, 1.59) | 37% | 1.55 (1.28, 1.87) | 8% | 1.21 (0.80, 1.84) | 17% | 1.27 (0.94, 1.71) |
| past week (min) | 150-299 | 15% | 1.18 (0.73, 1.91) | 43% | 1.79 (1.47, 2.17) | 9% | 1.35 (0.82, 2.23) | 18% | 1.29 (0.91, 1.83) |
|  | 300-449 | 11% | 0.94 (0.51, 1.71) | 40% | 1.62 (1.27, 2.05) | 6% | 1.01 (0.49, 2.11) | 19% | 1.44 (0.97, 2.13) |
|  | ≥450 | 17% | 1.33 (0.82, 2.14) | 49% | 2.20 (1.64, 2.95) | 7% | 1.00 (0.44, 2.32) | 19% | 1.31 (0.82, 2.09) |
| Cycling | None | 12% | 1 | 32% | 1 | 5% | 1** | 12% | 1 |
| for transport in | 1-149 | 16% | 1.03 (0.65, 1.64) | 33% | 0.79 (0.59, 1.07) | 22% | 1.95 (1.15, 3.31) | 35% | 1.22 (0.88, 1.68) |
| past week (min) | 150-299 | 24% | 1.16 (0.69, 1.96) | 46% | 0.95 (0.68, 1.31) | 39% | 2.85 (1.57, 5.19) | 41% | 1.05 (0.72, 1.55) |
|  | ≥300 | 18% | 1.07 (0.36, 3.13) | 53% | 1.35 (0.78, 2.32) | 29% | 1.98 (0.62, 6.29) | 59% | 1.47 (0.93, 2.31) |
| Cycling | None | 11% | 1* | 31% | 1 | 4% | 1*** | 10% | 1*** |
| for recreation in | 1-149 | 21% | 1.64 (1.07, 2.54) | 48% | 1.27 (1.01, 1.61) | 28% | 2.69 (1.56, 4.62) | 51% | 2.24 (1.72, 2.92) |
| past week (min) | 150-299 | 15% | 1.23 (0.52, 2.89) | 49% | 1.15 (0.79, 1.67) | 15% | 1.49 (0.57, 3.92) | 56% | 2.54 (1.77, 3.64) |
|  | ≥300 | 30% | 2.31 (1.12, 4.76) | 50% | 1.08 (0.73, 1.61) | 45% | 5.20 (2.07, 13.12) | 55% | 2.56 (1.66, 3.96) |

*p<0.05, **p<0.01, ***p<0.001 for heterogeneity. WT=walking for transport, WR=walking for recreation, CT=cycling for transport, CR=cycling for recreation, CI=confidence interval, RR=relative risk. Multivariable analyses adjusted for all variables in the column. † Cells merged with reference group because of small cell sizes (<5 Connect2 users).
